# Metabolic Structure: A Paradigm Revisited in Light of the Fundamental Parameters of Living Systems

**DOI:** 10.1002/bies.70193

**Published:** 2026-10-09

**Authors:** Jacques Fantini

**Affiliations:** ^1^ Aix‐Marseille Université (AMU) IHU Méditerranée Infection Microbes Evolution Phylogeny and Infections (MEPHI) Marseille France

**Keywords:** electrostatic potential, emergent properties, flux control coefficients, fundamental biological parameters, hepatic zonation, inherited metabolic diseases, lipid rafts, metabolic control analysis, Warburg effect

## Abstract

The concept of ‘metabolic structure’, which considers metabolism as a stable, genetically encoded architecture with enzymes occupying fixed control positions, is indispensable but incomplete, and it obscures metabolism's true nature. I propose redefining metabolism as a dynamic field of emergent biochemical fluxes, governed by six fundamental parameters: time, water, entropy, quantum effects, space, and electrostatic potential, shaping the thermodynamic trajectories of living matter. Metabolic control analysis already supports this view: flux control coefficients are state‐ and time‐dependent coordinates within a thermodynamic field, not intrinsic enzyme properties. This perspective manifests across scales: electrostatics govern fructose 1,6‐bisphosphate cleavage in aldolase; hepatic zonation shows spatial reorganization through a single parameter; glucose deprivation quantitatively redirects intestinal cancer cell metabolism from glycolytic to oxidative flux, driving differentiation into an enterocyte‐like state; post‐meal walking redirects glucose flux from storage to oxidation without pharmacological intervention; inherited metabolic diseases can sometimes be managed without correcting the causal mutation. These examples converge into a new framework: metabolism possesses not only a sophisticated architecture but also states that shape its fluxes. And states, unlike static structures, can be reconfigured.

## Graphical abstract

Metabolism is redefined as a dynamic thermodynamic field shaped by six fundamental parameters (time, water, entropy, quantum effects, space, and electrostatics) rather than fixed genetic architecture. Case studies from enzyme kinetics to cancer cell differentiation show metabolic states, not structure alone, determine flux and control, and can be reconfigured.

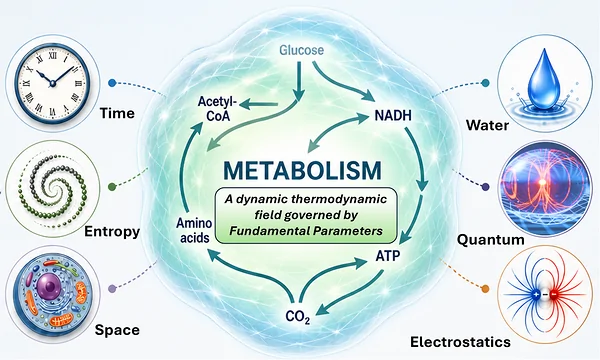

## 1 Introduction

For decades, metabolism has been taught in universities worldwide as a static map of biochemical reactions, presented as a fixed, rail‐map‐like network of metabolic pathways [1] to be memorized, reproduced for exams, and then forgotten (Figure 1). Biochemistry teachers must question this reductionism: students master enzyme names and stoichiometries for exams but acquire no usable biological insight. In essence, they learn metabolic pathways (which of course are important), but not what metabolism actually is (which should be the ultimate goal of teachers).

**FIGURE 1:**
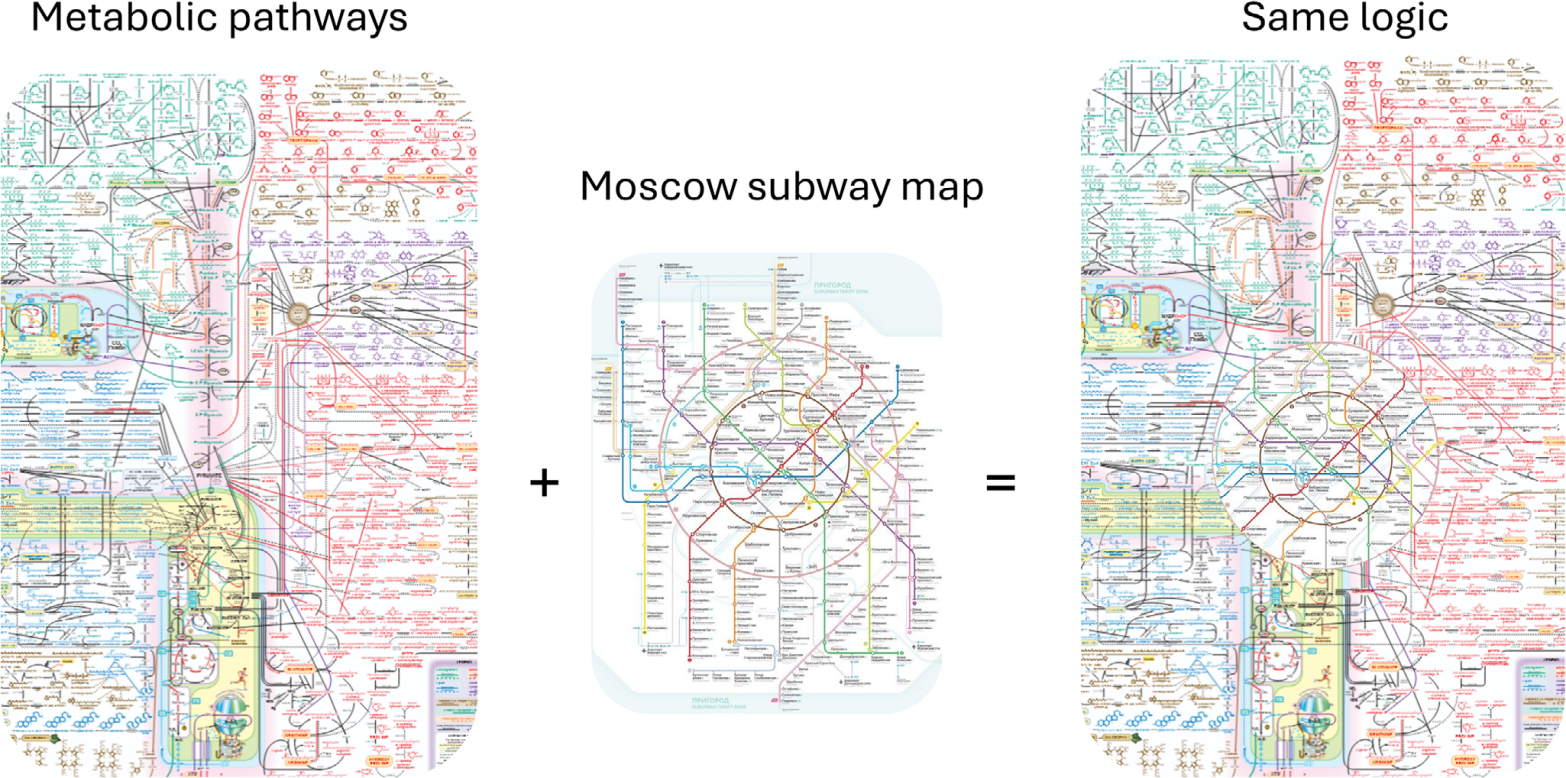
Rail map network representation of metabolism: a convergence of two independent organizational logics. Left panel: Human metabolic pathways, illustrating the scale‐free network topology of intermediary metabolism (source: Wiki‐media Commons, CC BY‐SA 4.0). Center panel: Moscow subway network map (source: Sameboat, Wiki‐media Commons, CC BY‐SA 3.0). Right panel: Superposition of both networks, labeled “same logic.” In both systems, a large number of directed connections link invariant nodes (metabolites or transit stations, respectively), generating hubs of high connectivity.

This pedagogical failure reflects a deeper conceptual problem: the discipline itself has long implicitly treated metabolism as a stable, hierarchical, and genetically encoded architecture in which specific enzymes occupy fixed control positions. The concept of a “rate‐limiting step” has become almost dogmatic, as if metabolic control resided permanently in a single enzymatic bottleneck [2], instead of being continually redistributed throughout the system according to thermodynamic constraints. Metabolic control analysis (MCA) challenged this view several decades ago by mathematically demonstrating that flux control coefficients sum to one, and by revealing (as a robust experimental finding rather than a deductive consequence of that theorem) that control is frequently distributed across multiple steps rather than residing permanently in a single rate‐limiting enzyme [3].

Here, I propose that the persistent notion of “metabolic structure” constitutes a convenient but misleading reification of what is, in reality, a dynamic landscape of emergent flux distributions. Drawing on the fundamental biophysical parameters of living systems recently formalized by our team (time, water, entropy, quantum mechanisms, space, and electrostatic potential [4]), I argue that metabolic organization at every scale, from the active site of an enzyme to the metabolic zonation of an organ, arises from the joint action of genetic and regulatory architecture and the six fundamental parameters, with the latter frequently underappreciated as an independent, quantifiable determinant of the realized state. This reinterpretation is integrated into a new framework, referred to as MSCFP (metabolic structure challenged by fundamental parameters).

The urgency of this conceptual reorientation is further illustrated by a widespread problem in contemporary metabolic research: the possible misinterpretation of metabolomic data [5, 6]. High‐throughput metabolomics platforms now generate vast inventories of metabolite concentrations, which are routinely used as proxies for metabolic activity and flux. However, metabolite concentrations and metabolic fluxes are fundamentally distinct quantities, a point that MCA established decades ago but that remains poorly understood outside the specialist community.

The manuscript is organized as follows: metabolic control is re‐examined through the lens of fundamental parameters; the electrostatic and configurational nature of metabolic energy is then examined, together with its effects on enzyme elasticities; spatial organization is illustrated through hepatic zonation; a quantitative, multiply‐testable case study of glucose‐ and field‐driven flux redistribution in intestinal cancer cells is then presented as a falsifiable test of the MSCFP framework; and clinical and therapeutic implications are discussed.

## 2 Fundamental Parameters of Biology as a Conceptual Framework

The conventional opposition between ‘structure’ and ‘function’ in biology obscures the underlying physical determinants that govern living systems [7]. In a more fundamental perspective, biological organization emerges from a restricted set of parameters—time, water, entropy, quantum mechanisms, space, and electrostatic potential—that define the accessible thermodynamic trajectories of matter under life‐compatible conditions.

The six fundamental parameters defined above are not independent axes but mutually entangled dimensions of a single physical reality [4]. (i) Time constrains all metabolic reactions: enzymes exist precisely to make biochemical processes compatible with the timescales of living systems, with rate accelerations reaching factors equivalent to billions of years of uncatalyzed reaction, as in uroporphyrinogen decarboxylation or phosphomonoester hydrolysis [8, 9]. (ii) Water is not a passive solvent but an active matrix that participates directly in biological dynamics [10, 11, 12, 13, 14, 15]. Fluctuations in water structure and dynamics modulate local energy landscapes [16, 17, 18, 19]. (iii) Entropy production is a necessary signature of irreversible processes and thus of metabolic activity, but it also implies permanent microstate turnover and configurational variability [20]. A central implication is that entropy is incompatible with any strict notion of fixed structure in living matter. (iv) Quantum mechanisms contribute at the level of discrete energy transfer, tunneling, and probabilistic state transitions, thereby influencing reaction rates, electron and proton transport, and molecular recognition at sub‐nanometric scales [21, 22, 23]. (v) Spatial organization through compartmentalization, membrane topology, and geometrical constraints [24, 25] defines diffusion pathways, reaction volumes, and encounter probabilities, thereby sculpting the effective connectivity of metabolic networks. (vi) Electrostatic potentials, arising from fixed and mobile charges in proteins, nucleic acids, lipids, ions, and water, mediate long‐range interactions that steer molecular encounters and pre‐organize reaction complexes well beyond direct contact distances [26, 27, 28, 29, 30]. These fields dynamically reshape energy landscapes in response to local changes in charge distribution, ionic strength, and hydration, coupling biochemical events over nanometer to subcellular scales. To make this framework directly operational, Table 1 associates each parameter with a measurable proxy and, where available, a worked example developed later in this article (Table 1, refs [31, 32, 33].) On this basis, metabolism can be redefined as a dynamic field of emergent biochemical fluxes, where the apparent organization of “metabolic structure” reflects instantaneous thermodynamic and electrostatic constraints rather than a fixed molecular architecture. The sections that follow examine how this framework, referred to as MSCFP, reinterprets canonical metabolic pathways as emergent flux solutions to time‐ and environment‐dependent thermodynamic problems.

**TABLE 1:** Measurable proxies for the six fundamental parameters of MSCFP.

Parameter	Measurable proxy	Worked example
Time	Reaction relaxation times; catalytic rate enhancement (kcat/kuncat)	Rate accelerations equivalent to billions of years of uncatalyzed reaction [8, 9]
Water	Water activity; hydration‐shell dynamics (dielectric relaxation, residence times)	In carbonic anhydrase, a zinc‐bound water molecule serves as the nucleophile attacking CO_2_, and the rate‐limiting step of turnover is proton transfer through an ordered intramolecular water network [31, 32]
Entropy	Entropy production rate (dissipation per unit time)	Hepatic zonation as a spatial entropy‐production gradient (Section 5)
Quantum effects	Kinetic isotope effects; rates of electron/proton tunneling	Experimental evidence provided by soybean lipoxygenase‐1, where hydrogen transfer displays a kinetic isotope effect far exceeding semiclassical predictions, together with a near‐temperature‐independent activation energy, hallmarks of a hydrogen‐tunneling mechanism [33]
Space	Diffusion distances; compartment volume; local effective concentrations	Hepatic zonation and HT‐29 differentiation (Sections 5, 6)
Electrostatic potential	Local surface potential; membrane potential; ionic strength; pH	Phosphate–phosphate repulsion in aldolase catalysis (Section 4)

## 3 Metabolic Control Analysis (MCA) Revisited

MCA was originally developed to quantify how perturbations in individual enzymatic steps affect steady‐state fluxes and metabolite concentrations in biochemical networks [34, 35]. In its classical formulation, control is expressed through flux control coefficients, which measure the relative sensitivity of a system‐level flux to small changes in the activity of a given enzyme [36, 37]. This formalism already challenged the notion of a single “rate‐limiting” step [34, 38], but its deeper implications become fully apparent when these coefficients are interpreted in the context of the six fundamental parameters introduced above.

Overall, the concept is based on flux control coefficients as global properties of the system at a given steady state [34, 39]. Flux control coefficients quantify how much each enzyme in a pathway contributes to controlling the flux through that pathway, expressed as the fractional change in flux caused by a fractional change in that enzyme's activity ($C_{\mathrm{Ei}}^{J}=\partial \ln J/\partial \ln u_{i}$) [35, 37]. In other words, a flux control coefficient measures the relative change in pathway flux in response to a relative change in a parameter (often an enzyme's activity), so by definition it is a sensitivity property of the whole system, not of the enzyme alone [40]. They embody the central insight of metabolic control analysis: that control of flux is a distributed, systemic property; the sum of all flux control coefficients in a pathway equals 1 [34, 41]. Control is frequently, though not universally, distributed across multiple steps in real metabolic networks; in simple linear pathways under certain kinetic regimes, a single enzyme can approach full control (coefficient ≈ 1) while others approach zero [37, 41, 42]. Explicitly introducing these coefficients is therefore mandatory if one wants to move beyond simplistic notions of rate‐limiting enzymes and correctly describe how metabolic pathways respond to genetic, pharmacological, or physiological perturbations at the systems level [43].

In networks where control is genuinely distributed, changes in one enzyme's kinetics, expression level, or surrounding environment can redistribute control among the steps; reactions operating close to thermodynamic equilibrium, by contrast, typically retain a negligible control coefficient regardless of such changes [40]. Experimental and theoretical studies clearly show that the same enzyme can have very different flux control coefficients under different conditions (substrate saturation, allosteric effectors, pathway branching, nonlinear kinetics), underscoring that control is a systemic property rather than a fixed characteristic of an enzyme [44, 45]. Yet it is important to note that the summation theorem does not, by itself, guarantee that control is shared among multiple steps, nor that nonzero control persists at every step. Distributed control—well documented experimentally in several systems [46, 47, 48]—is a property of specific network topologies and kinetics, not a deductive consequence of the theorem. Three distinct claims should not be conflated: (i) a flux control coefficient is a state‐dependent sensitivity of a steady‐state flux to enzyme activity; (ii) when the metabolic state changes, control coefficients may be redistributed across the network; and (iii) the system necessarily compensates for a targeted perturbation to restore flux or phenotype. MCA formally establishes (i) and accommodates (ii); it does not, on its own, establish (iii), which is a separate, system‐specific empirical claim. In practical terms, when control is genuinely distributed, targeting a single ‘rate limiting’ reaction may underestimate the cooperativity and reconfigurability of control in nonequilibrium systems [43].

Several experimental studies have directly demonstrated the redistribution of metabolic control in response to changing conditions [49, 50]. In the oxidative phosphorylation system of rat liver mitochondria, Groen et al. demonstrated that control over oxygen consumption is distributed between substrate supply, the respiratory chain complexes, and the ATP synthase, with the distribution varying markedly between resting and active states [46]. In human erythrocytes, the classic studies of Rapoport et al. established that no single enzyme controls glycolytic flux under all conditions, and that control coefficients change as a function of metabolite concentrations and energy demand [47]. More recently, top‐down MCA approaches applied to hepatocyte metabolism have shown that control over biosynthetic flux shifts from mitochondrial to cytosolic compartments depending on nutrient availability [48]. Together, these studies confirm that metabolic control is not a fixed property of individual enzymes but a dynamic, system‐level phenomenon that is continuously redistributed in response to environmental and physiological parameters.

When MCA is viewed through a thermodynamic lens, the dependence of control coefficients on entropy‐related constraints becomes explicit. In a nonequilibrium steady state, fluxes are driven by thermodynamic forces (affinities) and sustained by continuous entropy production [51]. Changes in the entropy production profile (for instance, through altered substrate supply, energy charge, or dissipation pathways) modify the effective driving forces and, consequently, redistribute control among enzymes [52]. I propose, as a conceptual extension rather than a derivation from classical steady‐state MCA, that state‐dependent redistribution of control may parallel—by analogy with nonequilibrium thermodynamic principles [51] and existing dynamic/transient formulations of control analysis [52]—a reallocation of dissipative load across the network. Formal treatment of control coefficients as continuous thermodynamic‐field coordinates remains beyond the scope of this Think Again essay and is flagged here as a hypothesis for future quantitative work.

Moreover, flux control coefficients are not only state dependent but also time dependent [52]. In transient regimes during developmental transitions, stress responses, or signaling events, the redistribution of metabolite pools, post translational modifications, and changes in protein–protein interactions lead to continuous temporal reconfiguration of control [53]. Even in systems that eventually reach a quasi–steady state, the path taken through phase space can involve transient windows in which different enzymes transiently dominate control [54]. This temporal variability gives operational content to the time parameter: control is not a static mapping from structure to function, but a dynamic field that evolves as the system explores its thermodynamic landscape [55, 56, 57].

Revisiting MCA under this extended conceptual framework reveals that metabolic control is not simply a property of enzymes arranged in a fixed network. Rather, it is a dynamic expression of how time, entropy production, water‐mediated organization, spatial compartmentalization, and electrostatic fields jointly sculpt elasticities and redistribute control across the network. In this sense, MCA provides a quantitative bridge between the microscopic parameters that define local reaction environments and the emergent, time dependent pattern of metabolic “structure” at the systems level.

## 4 Electrostatic Potential and Enzyme Elasticities

Where is potential energy actually stored in biomolecules? From decades of teaching experience, and as extensively documented in the biochemistry education literature [58, 59], students invariably answer “in chemical bonds.” This is conceptual inversion: bond breaking requires energy input. The net thermodynamic balance (ΔG°′), not any single bond, is the true locus of potential energy transduction. In this sense, “energy stored in bonds” is a pedagogically convenient fiction that obscures the electrostatic and configurational nature of molecular potential energy—the very quantity our framework places at the center of metabolic organization. The usable potential energy in biomolecules such as glucose or fatty acids resides in the reducing power of their C─H bonds, specifically in electrons held at high electrochemical potential by reduced carbons, whose transfer to O_2_ via the mitochondrial electron transport chain releases the free energy driving ATP synthesis. Bond‐breaking per se does not liberate biological energy; it is the vectorial transfer of electrons down a thermodynamic gradient, governed by electrostatic potential differences across molecular and membrane scales, that constitutes metabolic work [60, 61]. Glycolysis exemplifies this: far from mere cleavage, it strategically dehydrogenates to yield H‐enriched pyruvate (the methyl group of carbon‐3) (Figure 2A), upgrading carbohydrate poverty into a metabolite with high energy value. This dynamic, parameter‐driven adaptation is generally dismissed in conventional pedagogy but central to metabolism's rejection of rigid “structure.” As discussed below, this reframing of metabolic energy involves taking into account enzyme‐level electrostatic mechanisms, rather than a static bond‐energy accounting.

**FIGURE 2:**
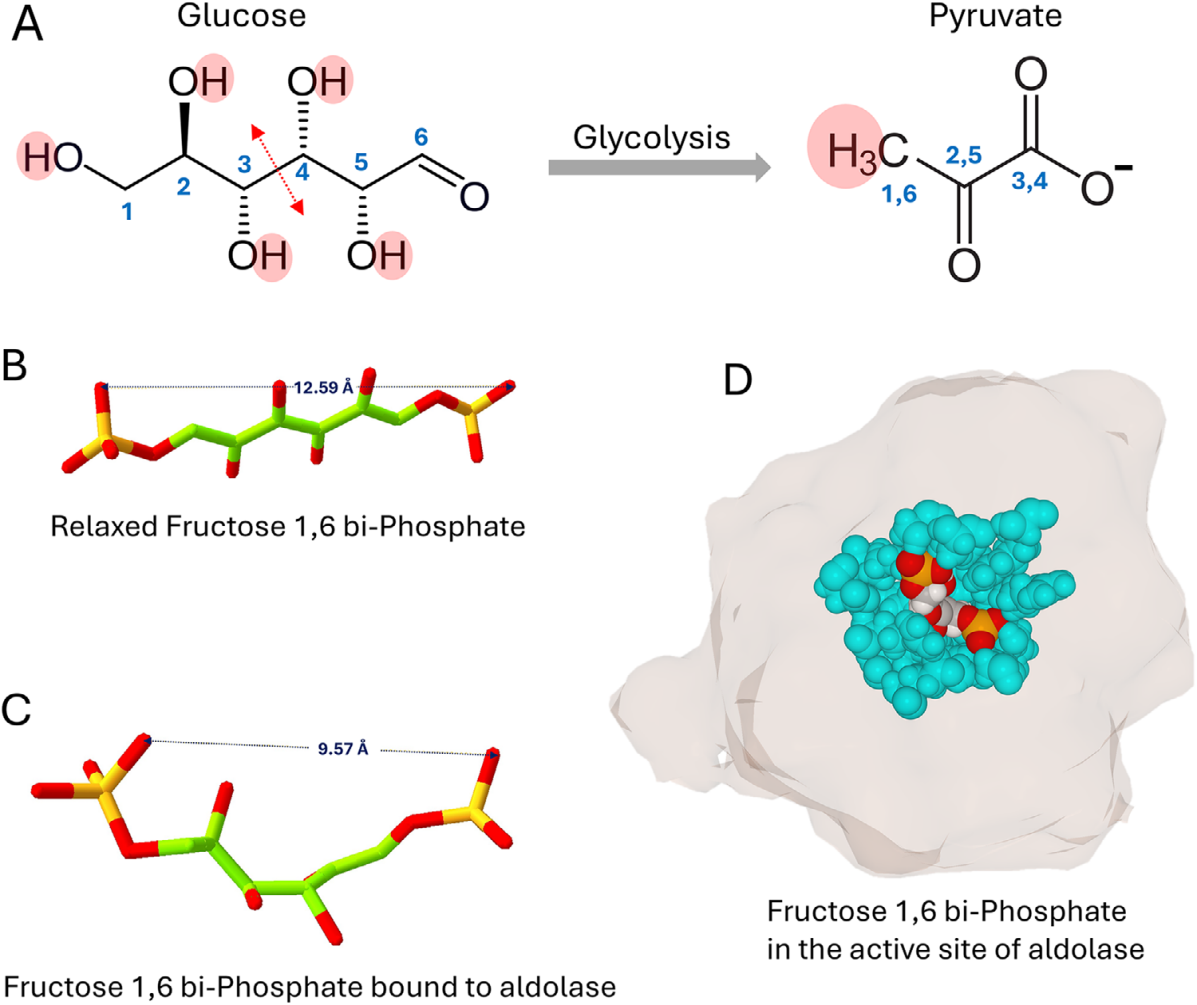
The biochemical “philosophy” of glycolysis grounded in the fundamental parameters of biology. (A) Glucose (C_6_H_12_O_6_) enters glycolysis as a C6 compound and emerges as two C3 compounds that are net oxidized (loss of 4e^−^, captured as 2 NADH) yet carry a hydrogen‐enriched methyl group, a counterintuitive outcome arising because glycolysis concentrates reducing power into the C3 methyl carbon. Carbon numbering of glucose and the corresponding atoms in pyruvate are indicated; the aldolase cleavage between C3–C4 is shown by the double red arrow. (B–D) The conformational change imposed by aldolase on its substrate illustrates how spatial and electrostatic parameters constraints govern catalytic efficiency. (B) Fructose 1,6 biphosphate in water (extended conformer). (C) Conformation of Fructose 1,6 biphosphate bound to aldolase. (D) Fructose 1,6 biphosphate in the active site of aldolase (pdb #4ALD). Inter‐phosphate distances were measured directly from the crystal structure of human muscle aldolase (PDB: 4ALD) and from the energy‐minimized conformation of free F‐1,6‐BP (computed using HyperChem/Polak–Ribière minimization).

Electrostatic fields generated by fixed and mobile charges in proteins, nucleic acids, lipids, and small ions extend over nanometric distances and pre‐organize the configurational space explored by substrates and cofactors [62]. Rather than acting only at the point of chemical transformation, electrostatic potentials influence the entire approach trajectory, orientational alignment, and stabilization of intermediate states [63, 64, 65]. In kinetic terms, these fields shape the enzyme elasticities, understood as local sensitivities of reaction rates to changes in substrate, product, or effector concentrations [42, 66]. Elasticities thus become context‐dependent quantities, determined not only by intrinsic catalytic parameters but also by the spatial and temporal structure of the surrounding electrostatic field.

A canonical example of how electrostatic potential governs long‐range molecular interactions operates in glycolysis itself: sequential phosphorylations at C1 and C6 (sequentially transforming glucose into Fructose‐1,6‐bi‐phosphate, abbreviated F‐1,6‐BP) generate mutual electrostatic repulsion that fragilizes the C3‐C4 bond, reducing aldolase activation energy (Figures 2B–D). This is biophysical catalysis: charge repulsion pre‐pays the bond‐breaking energy. Yet the enzyme lays an active role in this process [67]. When F‐1,6‐BP binds within the Class I aldolase active site, the enzyme imposes a specific constrained conformation on the substrate [68]. This is a well‐documented form of ground‐state destabilization [69, 70]. Indeed, this anchoring process forces the substrate into a geometry it would not spontaneously adopt in free solution.

Phosphate–phosphate electrostatic repulsion is thermodynamically sound. Indeed, the distance between the phosphate groups is approximately ∼12.6 Angstroms in solution (Figure 2B) versus ∼9.6 Angstroms in the active site of the enzyme (Figure 2C). This ∼3 Å reduction in inter‐phosphate distance upon active site binding is physically significant. The crystal structure of human muscle aldolase complexed with F‐1,6‐BP (PDB: 4ALD) confirms that the enzyme forces the molecule into a more compact conformation than it adopts in free solution (Figure 2D). This electrostatic contribution operates alongside the well‐established Schiff‐base formation and acid/base catalysis of class‐I aldolases [71]; ground‐state destabilization through charge repulsion and covalent catalysis act jointly to lower the activation barrier.

Electrostatic fields also modulate the apparent Michaelis constant and turnover number by reshaping the free energy landscape between bulk solution and the transition state [72]. Critically, small changes in charge distribution through phosphorylation, allosteric ligand binding, or local ionic fluctuations can shift elasticities without any modification of the underlying protein backbone, demonstrating that kinetic identity is in part encoded in the electrostatic microenvironment rather than in covalent structure alone [73, 74]. Since enzyme elasticities are shaped by local and global electrostatic fields, then flux control coefficients necessarily inherit the dynamics of these fields. In this perspective, the “metabolic structure” quantified by MCAnalysis is inseparable from the underlying electrostatic architecture of the cell, which continuously reorganizes in time and space [4, 75].

## 5 Hepatic Metabolic Zonation: Spatial Reorganization Through a Physicochemical Gradient

The metabolic heterogeneity of the liver parenchyma along the porto‐central axis, first systematically described and analyzed by Gebhardt [76], represents one of the most compelling natural demonstrations that spatial metabolic organization emerges from physicochemical gradients rather than from a pre‐encoded structural blueprint. Hepatic metabolic zonation can be defined as the differential distribution of metabolic functions along the porto‐central axis of the liver lobule [77, 78, 79]. In essence, it demonstrates how fundamental biophysical parameters impose spatial organization on biochemical fluxes without requiring genetic predetermination (Figure 3). The oxygen gradient, decreasing from approximately 60–65 mmHg in the periportal zone to 30–35 mmHg in the perivenous (centrilobular) zone, serves as the primary spatial organizer [80]. This gradient arises from the anatomical arrangement of sinusoids and the oxygen consumption of upstream hepatocytes, creating a continuous physicochemical axis that dictates zonal enzyme expression, substrate utilization, and detoxification capacities.

**FIGURE 3:**
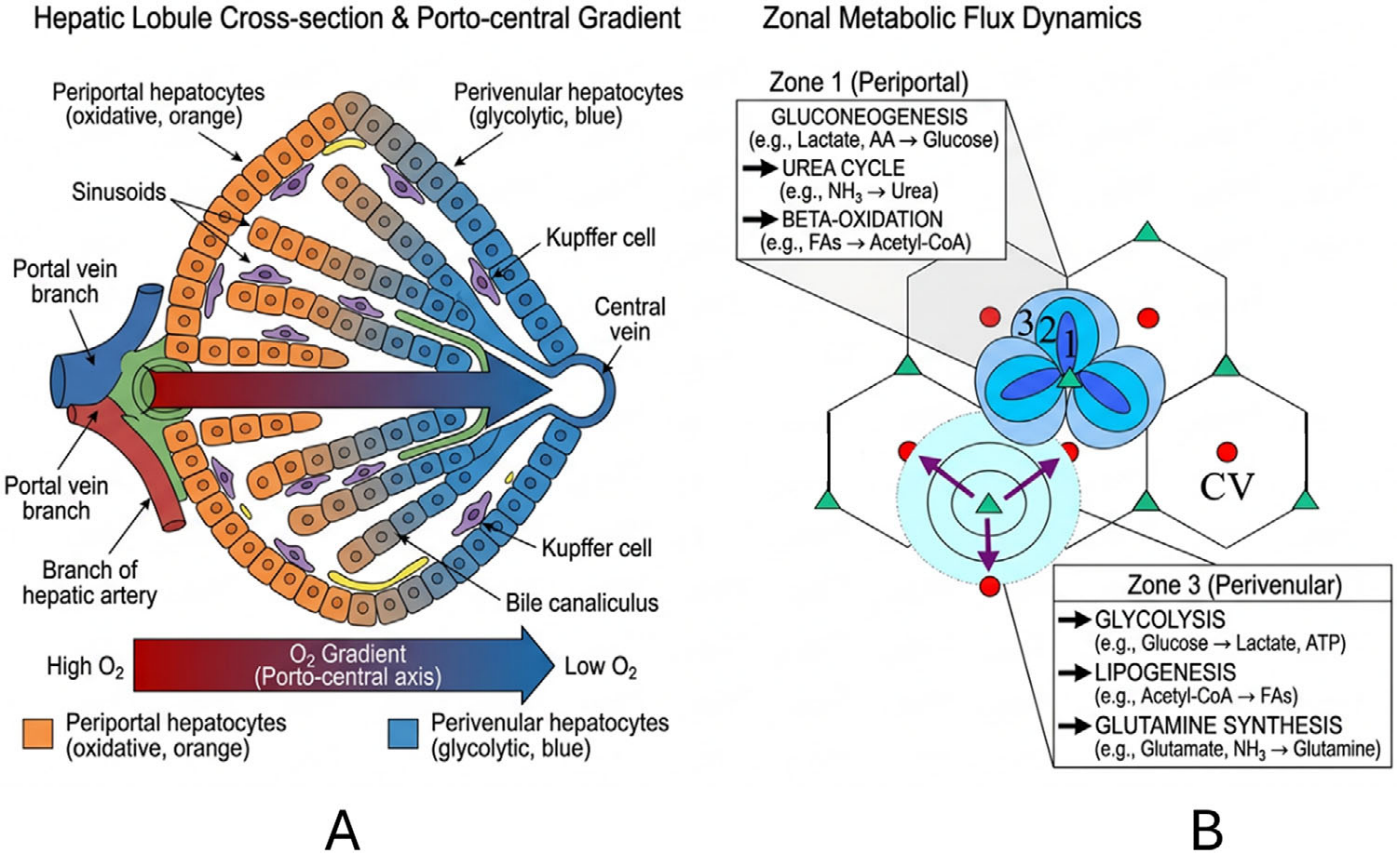
Hepatic metabolic zonation emerges from physicochemical gradients rather than genetic programming, as predicted by the MSCFP framework. (A) Schematic liver lobule cross‐section along the porto‐central axis: oxygen and nutrient gradients (red → blue) spontaneously generate periportal hepatocytes (orange; oxidative metabolism) and perivenous hepatocytes (blue; glycolysis/lipogenesis) without structural reprogramming. (B). The dynamics of metabolic fluxes determine three distinct zones of enzymatic activity (numbered 1 to 3), ranging from the periportal zone (zone 1) to the perivenous zone (zone 3), passing through a transition zone (zone 2). The metabolic signature of zones 1 and 3 is indicated.

Experimental reconstitution of this gradient on microfluidic chips confirms that oxygen alone is sufficient to establish zonation in primary hepatocyte cultures [81]. When hepatocytes are exposed to a linear O_2_ gradient mimicking physiological condition, they spontaneously adopt periportal (gluconeogenic, oxidative) versus perivenous (glycolytic, lipogenic) metabolic profiles, including differential expression of key enzymes such as phosphoenolpyruvate carboxykinase (PEPCK) and glutamine synthetase. This functional polarization occurs without exogenous morphogen gradients, underscoring that spatial organization emerges directly from the thermodynamic constraints imposed by the physical gradient.

Remarkably, more than 90% of hepatic metabolites exhibit spatially organized distributions along the porto‐central axis, reflecting the gradient's pervasive influence on flux partitioning [82]. Periportal hepatocytes, operating under higher oxygen availability, favor aerobic respiration, urea synthesis, and gluconeogenesis, while perivenous cells exploit lower oxygen tension for glycolysis, lactate production, and detoxification [79]. This metabolic division optimizes the liver's capacity to handle heterogeneous nutrient and toxin loads, with entropy production locally tuned to oxygen‐dependent respiratory limits. In thermodynamic terms, the oxygen gradient defines discrete metabolic zones as regions of space where distinct steady‐state fluxes minimize free energy dissipation under local constraints. Electrostatic potentials at enzyme active sites and transcriptional complexes integrate these spatial cues into adaptive responses, ensuring that the liver's global function emerges from the coherent superposition of local parameter‐driven behaviors [83]. Zonation thus illustrates a central tenet of MSCFP: metabolic organization is not fully specified by architecture alone but is co‐determined by time‐varying physical fields acting on that architecture. We note this example is presented as an illustrative analogy consistent with MSCFP rather than a quantitative test of the framework (see Section 6 for the latter).

## 6 A Convergent, Multiply‐Testable Case for MSCFP: Glucose Deprivation and Flux Redistribution in HT‐29 Colon Cancer Cells

A central requirement for any scientific framework is that it generates directional, quantitative predictions tested against experimental data, rather than retrospective reinterpretations of known phenomena. We address this requirement using the HT‐29 human colon adenocarcinoma model. Under glucose‐replete conditions, undifferentiated HT‐29 cells sustain aerobic glycolysis: ∼85% of glucose carbon is converted to lactate, with only ∼15% of acetyl‐CoA entering the TCA cycle, while cells remain proliferative and lack enterocytic markers (brush border, tight junctions, sucrase‐isomaltase) [84, 85]. Quantitative ^1^ ^3^C‐tracer NMR flux analysis of the related HT29‐D4 clone [86] confirmed this redistribution directly: the fraction of labeled acetyl‐CoA entering the TCA cycle rose from 15% to 30% under glucose labeling, and from 55% to 85% under acetate labeling, in differentiated versus undifferentiated cells—a measured, directional doubling of oxidative flux driven by a single boundary‐condition parameter change, glucose withdrawal [87].

Within MSCFP, this is precisely the predicted signature of a field‐driven state transition: removing glucose reconfigures glycolytic and mitochondrial elasticities, consistent with a redistribution of flux control away from glycolysis toward oxidative phosphorylation and permitting the spatial reorganization (cytoskeletal, junctional) required for enterocyte‐like polarization [88]. Establishing this rigorously would require dedicated perturbation experiments or a kinetic model from which control coefficients could be calculated, which we flag as a natural next step. Yet this prediction is falsifiable in the strict sense, because it does not depend on glucose withdrawal alone. Indeed, independent field‐level interventions such as chronic suramin treatment (blocking autocrine IGF‐1/IGF‐1R signaling that sustains glycolytic flux) [89, 90] and treatment with the anti‐IGF‐1R antibody α‐IR3 [91], converge on the same glycolytic‐to‐oxidative switch and the same differentiated, enterocyte‐like phenotype [92], despite acting through unrelated molecular mechanisms (Figure 4). This convergence of independent perturbations onto a single predicted metabolic state is difficult to reconcile with a purely genetic, structure‐fixed view of metabolism, and constitutes the kind of quantitative, multiply‐testable prediction requested for a falsifiable MSCFP framework. In this respect, this model is a concrete illustration of a general principle: that metabolic reorganization in a pathological context follows parameter gradients rather than disease‐specific genetic programs. Indeed, similar observations have been made in human tumor cells of various tissue origins [93, 94, 95, 96, 97, 98]. Mechanistically, this link between flux and morphology operates through the space parameter: high glycolytic flux generates lactate and protons beyond the buffering capacity of the cells, producing a sustained intracellular acidosis that protonates key residues in cytoskeletal proteins [99, 100], destabilizing the spatial self‐organization required for apical‐basal polarity, tight junctions, and brush border assembly. Suramin and α‐IR3 act precisely by lowering glycolytic flux and normalizing intracellular pH [90, 91], thereby releasing (rather than instructing) the intrinsic capacity of the cell for spatial self‐organization. Glucose withdrawal is not itself one of the six fundamental parameters; rather, it acts as a boundary condition that lowers electron/carbon input into the network, thereby altering the local entropy production rate and the electrostatic (redox) potential across glycolytic and mitochondrial compartments; i.e., it operates through the entropy and electrostatic parameters already defined in Section 2, rather than introducing an independent variable. The metabolic field is the obstacle; removing it is sufficient. We present this case as a quantitative, field‐level test of MSCFP, explicitly distinguished from the framework's other, illustrative analogies (hepatic zonation in Section 5, post‐meal walking in Section 8), which are not offered as quantitative tests of the framework.

**FIGURE 4:**
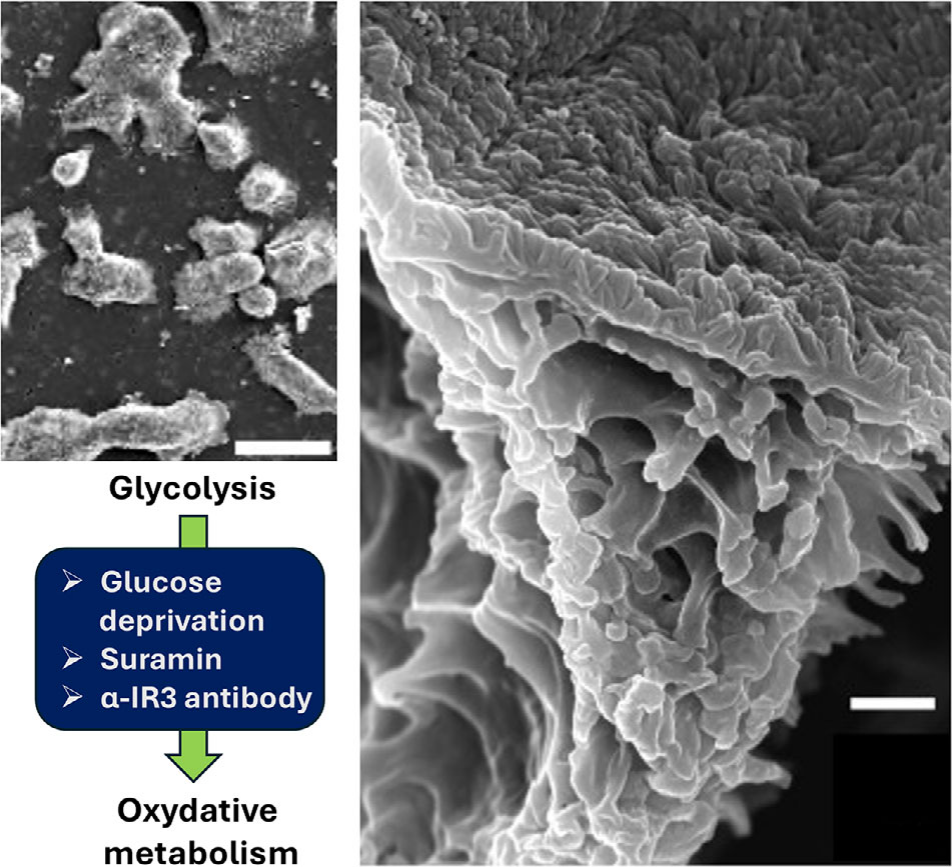
Metabolic reprogramming induces HT29‐D4 differentiation through multiple field‐level interventions, as predicted by the MSCFP framework. Scanning electron microscopy reveals a glycolytic‐to‐oxidative metabolic switch that transforms proliferative morphology into differentiated, enterocyte‐like states. (Left) Undifferentiated cells under glycolytic conditions (scale bar: 5 µm). (Right) Differentiated cells under oxidative metabolism, showing polarization, extensive microvilli, and increased surface complexity typical of enterocyte‐like polarization (scale bar: 2 µm). Interventions driving this transition include glucose deprivation, chronic suramin treatment, or the α‐IR3 anti‐IGF‐1 receptor antibody, each reconfiguring the metabolic field to favor differentiation over proliferation. Reproduced from [92] with permission from Elsevier.

## 7 Inherited Metabolic Diseases and the “Target the Target, Not the Arrow ” Paradigm: Gene is the Arrow, the Pathological Field is the Target

The MSCFP framework developed in this article appears, at first glance, to conflict with the reality of inherited metabolic diseases. If a genetic lesion abolishes an enzyme, no parameter manipulation can restore the missing sequence—CRISPR‐Cas9 would seem to be the only logically consistent strategy.

This apparent conflict dissolves the moment one distinguishes between the origin of a perturbation and the mechanism by which it generates a clinical phenotype. A genetic defect is a fixed modification of one molecular component. The clinical syndrome does not emerge from the lesion itself—it emerges from the downstream parameter imbalances the lesion sustains: substrate accumulation above a toxic threshold, product depletion below a functional threshold, disrupted concentration gradients that redistribute flux control coefficients across tissues. The gene is the arrow. The pathological field it generates is the target. The evidence is the foundation of clinical metabolic medicine. In each case below, the genetic lesion remains the causal origin of the disease; what these examples demonstrate is that the clinical phenotype can be managed by intervening on the downstream parameter gradient the lesion sustains, without correcting the lesion itself.

Phenylketonuria has been managed for six decades by dietary normalization of plasma phenylalanine, the substrate gradient parameter whose disruption—through competitive inhibition of the LAT1 transporter at the blood‐brain barrier—generates the neurological phenotype [101]. Lactose intolerance resolves completely on a lactose‐free diet, with zero action on the LCT gene [102]. Gaucher disease, caused by lysosomal β‐glucocerebrosidase deficiency, is controlled by substrate reduction therapy with eliglustat (normalizing the glucosylceramide biosynthetic gradient) [103], and pharmacological chaperone therapy with ambroxol (stabilizing the mutant protein's conformational field in the endoplasmic reticulum and recovering its lysosomal trafficking) [103, 104]. Neither of these strategies corrects the GBA1 gene; both are clinically validated. Apnea of prematurity, a temporal mismatch between an inhibitory adenosinergic field and an immature respiratory network, is resolved by caffeine's antagonism of A1 and A2A adenosine receptors, with documented long‐term neurodevelopmental benefits [105, 106].

The summation theorem of MCA provides the formal basis for this principle and simultaneously resolves a population genetics paradox that classical biochemistry has never fully explained: why do the majority of loss‐of‐function variants in metabolic enzymes remain clinically silent in heterozygous carriers? Because flux control is often distributed unevenly across a pathway, a step with a low flux control coefficient can tolerate a substantial reduction in enzyme activity (for example a 50% loss) without a proportional drop in pathway flux, since most of the flux is already governed by other, more limiting steps rather than requiring any redistribution [107]. The heterozygous carrier's metabolic field compensates for the genetic lesion without instruction and without knowing a gene has been lost. This intrinsic buffering capacity defines the operating range within which parameter‐level interventions are effective. The genetic lesion is present; the parameter field absorbs it; no phenotype emerges.

This buffering is not unlimited. When lesion severity exceeds the range of thermodynamic compensation, as in severe urea cycle defects or neuronopathic lysosomal storage diseases, where substrate accumulation causes irreversible structural damage before intervention is possible, gene correction addresses the root cause more efficiently than any downstream parameter manipulation. CRISPR is the right tool when the arrow cannot be left in place [108]. But for a large and underappreciated category of inherited metabolic diseases, the arrow can remain entirely in place. The pathological phenotype emerges from a parameter imbalance that is directly accessible to dietary, pharmacological, or environmental intervention, so that phenotypic rescue through parameter‐level management is not a compromise pending gene therapy. It is, in the MSCFP framework developed throughout this article, the correct level of intervention.

## 8 Practical Implications: A Drug‐Free Metabolic Flux Redirection in Corticosteroid‐Treated Patients

Chronic corticosteroid therapy provides a clinically relevant illustration of the MSCFP framework's therapeutic implications. Glucocorticoid excess is well established to inhibit insulin‐stimulated GLUT4 translocation to the plasma membrane in skeletal muscle [109], while simultaneously promoting GLUT4‐dependent glucose uptake and lipogenesis in adipocytes [110]. This differential tissue response redirects postprandial glucose flux toward fat storage, driving the characteristic weight gain associated with corticosteroid treatment [111]. In essence, this is not a structural metabolic defect; it is a parameter‐governed flux redistribution between two competing tissue sinks—a canonical illustration of the MSCFP principle that metabolic organization reflects a dynamic field state rather than a fixed enzymatic architecture [111, 112].

A simple, drug‐free intervention can reconfigure this field: a 30 min walk immediately after each meal activates insulin‐independent GLUT4 translocation to the sarcolemma of contracting skeletal muscle via AMP‐kinase and contraction‐stimulated signaling pathways, effectively outcompeting adipose tissue uptake for circulating glucose. Post‐meal walking has been shown to significantly reduce postprandial glucose AUC and redirect substrate utilization toward muscle glycolysis through a physicochemical parameter (mechanical field, oxygen demand, AMP/ATP ratio) what no pharmacological intervention targeting individual enzymes can accomplish with equivalent simplicity and safety [109, 110, 113]. This example embodies the MSCFP principle in its most translatable form: the target is not the enzyme, not the receptor, not the transporter: the target is the field. And the field can be reconfigured by walking.

Importantly, it should be emphasized that this example is provided solely for illustrative purposes within the MSCFP conceptual framework. Any physical exercise program in corticosteroid‐treated patients must be individually assessed and strictly supervised by qualified healthcare professionals, given the complexity of the underlying pathology and the potential contraindications associated with chronic glucocorticoid therapy. Finally, the beneficial effect of post‐meal walking on postprandial glucose regulation is not restricted to corticosteroid‐treated patients: Hashimoto et al. demonstrated that even a 10 min walk immediately after glucose intake significantly reduces postprandial glucose levels in healthy sedentary individuals [113], suggesting that parameter‐field reconfiguration through physical activity represents a universal metabolic intervention applicable to the broader population of physically inactive modern subjects.

## 9 Conclusion and Perspectives

What do we mean by “understand” metabolism? If we follow the argument developed throughout this article, it means recognizing that the biochemical reactions we observe are not the fundamental level of description. They are the visible surface of a deeper reality: a dynamic field of physicochemical constraints, organized by six parameters (time, quantum effects, water, entropy, space, and electrostatic potential) whose interplay continuously shapes the accessible thermodynamic trajectories of living matter. What we have been calling “metabolic structure” is the shadow this field casts onto the biochemical plane, a shadow that changes whenever the field changes, without requiring a genetic instruction.

The evidence assembled in this article converges with this conclusion. Metabolic control analysis shows mathematically that flux control is distributed, state‐dependent, and time‐dependent. There is no fixed rate‐limiting step, only instantaneous configurations of a thermodynamic field. This principle is both conceptual and therapeutic. Conceptually, it demonstrates that metabolic organization at every scale, from the active site of aldolase to the complex zonation of a hepatic lobule, is an emergent property of parameter gradients, not a structural blueprint. Therapeutically, it establishes that effective metabolic intervention cannot be achieved exclusively by targeting individual enzymes or blocking individual signaling steps. An enzyme targeted in isolation may have its effect compensated by redistribution among the remaining steps in networks where control is genuinely distributed. This is not universally a pharmacological failure: distributed control—a common but not universal property, well documented in several systems (see Section 3)—can lead single‐target interventions to be compensated by redistribution among remaining steps, contributing to phenomena such as drug resistance in specific, well‐characterized cases. Moreover, targeting individual metabolic enzymes carries inherent risks: many glycolytic and mitochondrial enzymes are moonlighting proteins with multiple, context‐dependent biological functions beyond their canonical catalytic roles [114, 115]. Inhibiting hexokinase or pyruvate kinase to modulate cancer metabolism, for instance, risks disrupting non‐metabolic functions in healthy tissues, generating unpredictable off‐target effects. Modulating the parameter gradient, for example through controlled glucose deprivation or ketogenic interventions, achieves the same flux redistribution without the risks associated with direct enzyme inhibition, because it acts on the field that governs all enzymes simultaneously rather than on a single molecular target.

Beyond theoretical biochemistry, this reframing has direct practical value, as in weight regulation, where it favors acting on the hormonal and thermodynamic milieu governing substrate partitioning (Section 8) rather than restricting caloric intake step by step. The MSCFP framework must also account for inter‐organ competition for metabolic substrates, a dimension of whole‐body metabolism that profoundly shapes systemic flux distributions. Organs and tissues are not metabolically equal: they differ in their parameter landscapes, their energetic demands, and critically, their competitive priority for shared substrates. The brain exemplifies this metabolic hierarchy with particular clarity: it is a selfish organ that maintains preferential access to glucose at the expense of peripheral tissues [116]. During prolonged fasting, the progressive shift from glucose to ketone body utilization in peripheral tissues is not merely an adaptive response: it is a parameter‐driven redistribution of substrate flux that preserves cerebral glucose supply while redirecting hepatic ketogenesis to meet the energetic demands of other organs [117]. Viewed through the MSCFP lens, whole‐body metabolic regulation emerges as a competitive parameter landscape in which organ hierarchies determine the direction and magnitude of substrate fluxes at the systemic scale.

The MSCFP framework is an operational scientific approach with three defining features. First, its method is explicit: any metabolic system is analyzed by systematically mapping the six fundamental parameters (time, quantum effects, water, entropy, space, and electrostatic potential) onto the system under study, replacing the classical enzyme‐centric or gene‐centric description with a parameter‐gradient description. Second, its workflow is reproducible: identify the process, determine the governing parameter(s), quantify the gradient, and predict the direction and magnitude of reorganization. Third, the framework generates testable predictions: if flux control coefficients are field‐dependent rather than structurally fixed, then experimentally modifying a fundamental parameter (e.g., altering the electrostatic environment of a receptor, modifying cellular hydration, or perturbing entropy gradients) should produce predictable, quantifiable shifts in metabolic flux, within the constraints set by an individual's genetic and physiological background, which determines the baseline elasticities on which parameter changes act. These predictions are experimentally accessible and falsifiable, distinguishing MSCFP from a mere conceptual metaphor. A first quantitative test of this kind has been presented in Section 6 [87], reinforced by convergent evidence from three independent interventions (glucose deprivation, suramin, α‐IR3), together with a proposed prospective reversibility test; full quantitative formalization linking the six parameters to flux control coefficients remains an objective for future work.

Finally, the MSCFP framework does not render classical biochemistry obsolete: it recontextualizes it. Enzymes, pathways, and control coefficients remain essential descriptors; MCA remains the indispensable quantitative tool for measuring how control is distributed at any given moment. What changes is the interpretive frame: these measurements describe snapshots of a moving field, not properties of a fixed architecture. The enzyme is not the cause. The field is the cause. And the field, constituted by time, quantum effects, water, entropy, space, and electrostatic potential—in essence the fundamental parameters of living matter—is the target. Metabolic structure alone does not determine metabolic state or control. What determines function is the state occupied within that structure. And states, unlike rigid structural assignments, can be reconfigured.

## Author Contributions

J.F. conceived the rationale, critically re‐analysed the published data and models, developed the alternative interpretation, and wrote the manuscript.

## Funding

The authors have nothing to report.

## Conflicts of Interest

The authors declare no conflicts of interest.

## Data Availability

The authors have nothing to report.
